# Maize (*Zea mays* L.) Productivity and Nitrogen Use Efficiency in Response to Nitrogen Application Levels and Time

**DOI:** 10.3389/fpls.2022.941343

**Published:** 2022-07-01

**Authors:** E. M. S. Gheith, Ola Z. El-Badry, Sobhi F. Lamlom, Hayssam M. Ali, Manzer H. Siddiqui, Rehab Y. Ghareeb, Mohamed H. El-Sheikh, Jebril Jebril, Nader R. Abdelsalam, Essam E. Kandil

**Affiliations:** ^1^Agronomy Department, Faculty of Agriculture, Cairo University, Giza, Egypt; ^2^Department of Plant Production, Faculty of Agriculture Saba Basha, Alexandria University, Alexandria, Egypt; ^3^Botany and Microbiology Department, College of Science, King Saud University, Riyadh, Saudi Arabia; ^4^Plant Protection and Biomolecular Diagnosis Department, Arid Lands Cultivation Research Institute, The City of Scientific Research and Technological Applications, Alexandria, Egypt; ^5^Plant Production Department, Arid Lands Cultivation Research Institute, City of Scientific Research and Technological Applications (SRTA-City), Alexandria, Egypt; ^6^Department of Agronomy, Kansas State University, Manhattan, KS, United States; ^7^Agricultural Botany Department, Faculty of Agriculture Saba Basha, Alexandria University, Alexandria, Egypt

**Keywords:** maize, nitrogen use efficiency, rates, timing, splitting, yield components

## Abstract

Productivity of maize (*Zea mays* L.) and nitrogen use efficiency (NUE) as affected by nitrogen application levels and timing were studied. The experimental design was a three-replication randomized complete block design (RCBD). The first factor was nitrogen levels (122, 240, 288 and 336 kg N/ha) and the second factor was nitrogen timing (50% of N at sowing and 50% of N before the first irrigation; T_1_, 50% of N at sowing and 50% of N before the second irrigation; T_2_ and 50% of N before the first irrigation and 50% of N before the second irrigation; T_3_). Results indicated that plant height, ear length, kernel weight, number of grains/rows, number of grains/ear and grain yields all increased significantly as nitrogen levels increased and the level of 336 kg N/ha significantly exhibiting the highest values in both seasons. In terms of nitrogen application time, maize yield parameters such as plant height, ear length, kernel weight/ear, number of grains/rows, number of grains/ear and grain yield were significantly affected by nitrogen timing, with the highest values obtained at T3 while the lowest values obtained at T1 in both seasons. The interaction had a significant impact on plant height and grain yield/ha, with the tallest plants, the highest yields and its components observed at 336 kg N/ha, with 50% of N applied during the first irrigation and 50% of N applied during the second. Furthermore, under the study conditions, NUE decreased dramatically as nitrogen levels increased and increased significantly as nitrogen application time changed.

## Introduction

Nitrogen (N) is one of the most essential plant nutrients. It is an essential component of a wide range of biological chemicals that play critical roles in photosynthetic activity, and agricultural productivity. Nitrogen availability can influence maize plant growth and grain yield. The influence of nitrogen availability on maize grain yield can be determined using physiological components such as the interception and effective use of radiation, as well as nitrogen partitioning to reproductive organ (Sandhu et al., 2021). Nitrogen fertilizer affects maize dry matter production by influencing leaf area development, maintenance, and photosynthetic efficiency (Kaur et al., 2012; Shah et al., 2021a). Nitrogen is critical for increasing soil production and crop efficiency (Habtegebrial et al., 2007). Applying more nitrogen to maize resulted in maximum emergence as well as improved plant elongation and yield (Keskin et al., 2005; Siddiqui et al., 2006). Nitrogen fertilizer also increased maize grain production (43–68%) and biomass (25–42%) (Ogola et al., 2002). N is also required for physiological and metabolic functions (Vijayalakshmi et al., 2013).

Maize (*Zea mays* L.) is an important cereal and multifunctional crop in the Poaceae family, it is used in human food, animal and poultry feed, and in industry for a variety of purposes including maize starch, dextrose, maize syrup, and maize flakes (Gul et al., 2021). It also grows well in a wide range of soil and climatic conditions. It extracts more nutrients than other crops such as tiny grain cereals and grain legumes. Maize is farmed for a variety of purposes, including animal feed (silage and grains), poultry feed (grains), and pigs feed (grains), as well as human consumption in the form of grains, sweet maize, and grain maize. In terms of acreage and output, it is Egypt’s third most significant basic food crop after wheat and rice. Maize is grown on 1.03 million hectares in Egypt, accounting for around 25.2 percent of all cultivated agricultural land, with an average yield of 8.3 tons per hectare (FAOSTAT, 2020). The Egyptian government hopes to reduce the gap between consumption and production by increasing grain production per unit area of agricultural land.

There are several ways to improve agricultural production, such as upgrading farming techniques, combining technology, and using new and high-yielding maze hybrids that are more efficient at consuming nitrogen and respond better to high nitrogen fertilizer rates to produce more grains. Nevertheless, nitrogen fertilizer is one of the most significant variables for crop development, high yield, its components, and quality, as nitrogen is needed to produce a variety of chemicals including chlorophyll and several enzymes. As a result, several researchers have discovered that increasing nitrogen availability improves maize yield (Oraby et al., 2005; Mansour, 2009; Rasmussen et al., 2015; Wasaya et al., 2017; Gheith et al., 2018; Bashir et al., 2021; Shah et al., 2021b).

According to the effect of nitrogen fertilization on maize plants, Amanullah et al. (2009) found that increasing of N rate caused accumulate in maize crops to heat units (thermal time) for tasseling, silking, and physiological maturity, and vice versa, in addition an increase in leaf area per plant, plant height, ear height and biomass yield. Also, other studies showed that when growing maize at high density with a 50% higher nitrogen rate (180 kg/ha) than the optimum rate (120 kg/ha) in 4–5 splits may result increased leaf area and plant height, resulting in maximum biological yield and thus increased maize crop production (Amanullah et al., 2009).

Furthermore, Mohkum Hammad et al. (2011) demonstrated that applying 250 kg N/ha nitrogen fertilizer resulted in the maximum plant height, number of kernels/cob, grain yield and its components. On the other hand, the highest biological yield was 300 kg N/ha. They also found that applying N in three splits at a rate of 250 kg N/ha yields the highest grain yield. In most yielding features, the S.C.10 outperformed other hybrids. When N was applied at a rate of 360 kg N/ha, the number of grains/rows, number of grains/ears, grain yield, and grain protein were all significantly higher. In the other study, a rate of 30 kg N/ha produced significantly more grain at 30, 45, 60, and 75 days after sowing, indicating that reducing N losses from the soil and making efficient use of N throughout important growth and development stages of maize would be more cost effective (Adhikari et al., 2016). Furthermore, yield-related variables like ear length, diameter, number of kernel/rows, number of kernels/rows, and test weight were highest at 120 kg/ha. The maximum yield was obtained at a rate of 120 kg N/ha (Karki et al., 2020). Plant height, ear length, ear weight, kernel yield, and number of kernels/ear, were all affected significantly by N application rates and timing (Gul et al., 2021).

Recently, high-yielding modern maize hybrids have been shown to accumulate more biomass and respond best to fertilizer. This, combined with increased photosynthesis capacity, has been linked to increased grain yield and nitrogen use efficiency, which reduces the rate of fertilizer such as nitrogen (N), lowering costs, as fertilizer prices rise. In this regard, the study was carried out to determine the most appropriate nitrogen rate.

## Materials and Methods

### Experimental Site

During the 2019 and 2020 summer seasons, two field experiments were conducted on a clay soil in texture at the Agriculture and Experiments Station at Giza, Faculty of Agriculture, Cairo University, Egypt, to investigate the impact of levels of nitrogen and timing on maize (*Zea mays* L. *cv* SC10) productivity and nitrogen use efficiency (NUE).

### Soil Analysis

Soil samples were collected from different locations at a depth 0–60 cm before sowing and analyzed some physio-chemical characteristics in the Research Center Laboratory, Faculty of Agriculture, Cairo University during both seasons. The soil texture was determined using hydrometer method (Topp et al., 1993). Organic matter was determined by the modified Walkey-Black method as suggested by Nelson and Sommers (1996). Available phosphorus (P) and potassium (K) were determined by the method of Olsen and Sommers (1982). Nitrogen was estimated according to Jackson (1958). Some physical and chemical analyses of the experimental site are presented in Table 1.

**TABLE 1:** Some physical and chemical analysis of the experimental site in 2019 and 2020 seasons.

Mechanical analysis			Chemical analysis		
Soil properties	1st Season	2nd Season	Soil properties	1st Season	2nd Season
Clay (%)	40.4	40.0	Available N (ppm)	29.3	32.5
Silt (%)	24.3	24.0	Available P (ppm)	10.6	11.3
Sand (%)	35.3	36.0	Available K (ppm)	265.5	267.5
Organic matter (%)	1.5	1.4	P*^H^*	7.9	7.8
Texture Class	Clay		EC (dS/m)	2.6	2.8

### Experimental Design

The two experiments were designed as factorial (two factors) and distributed in randomized complete block (RCBD) with three replications during the two seasons.

The first factor was different nitrogen levels, and the second factors was nitrogen application times. Each experimental plot area was 10.5 m^2^ (10.5 m^2^ = 1/400 feddan where one feddan = 4,200 m^2^ = 2.4 ha) and consisted of five ridges that were 3.0 meters long and 70 centimeters apart in the 2019 and 2020 seasons.

Each experiment had 12 treatments, each consisting of a combination of four nitrogen levels (N_1_ = 192, N_2_ = 240, N_3_ = 288, and N_4_ = 366 kg N/ha) and three application periods (T_1_; 50% of N at sowing and 50% of N before first irrigation, T_2_: 50% of N at sowing and 50% of N before second irrigation and T_3_: 50% of N at first irrigation and 50% of N at second irrigation).

Maize hybrid (Single Cross 10 = SC10) was planted on the 15th and 21st of May 2019 and 2020, respectively. Each hill had two kernels planted by hand method at 25 cm. Plants were thinned to one plant per hill before the first irrigation. The initial irrigation was provided 3 weeks after seeding, with further irrigations applied every 2 weeks throughout the growth season. The urea (46% N) levels were divided into two doses and applied according to the treatments. All other agronomic practices were kept normal and consistent for all treatments as recommended by the Ministry of Agriculture and Land Reclamation except those under study.

### Meteorological Data

The monthly of the meteorological data recorded during the cropping period (May to October) at Giza location (Altitude: 19 m. Latitude: 30.05°N Longitude: 31.21°E), Egypt during seasons of 2019 and 2020. The monthly average of meteorological data recorded during cropping period (May to October in 2019 and 2020 seasons) at the Meteorological Station of Agricultural Research Center, Ministry of Agriculture, Giza, Egypt is presented in Table 2 according to Muñoz and Grieser (2006).

**TABLE 2:** Mean temperature (C°), relative humidity (%) and rainfall (mm) at Giza, Egypt during 2019 and 2020 seasons.

Month	Temperature			Relative Humidity	Rainfall
		
	Min.	Max.	Aver.		
**Season 2019**					
May	17.0	34.2	25.6	43.7	0.0
June	17.8	34.5	26.2	55.0	0.0
July	19.6	34.1	26.9	62.0	0.0
August	20.0	34.3	27.2	65.0	0.0
September	17.9	32.3	25.1	69.7	0.0
October	16.5	30.0	25.7	68.0	0.0
Average	18.1	33.2	18.1	60.0	0.0
**Season 2020**					
May	16.2	31.8	23.5	48.0	0.0
June	18.1	34.6	26.8	56.0	0.0
July	20.0	34.2	27.2	61.0	0.0
August	20.1	34.4	27.3	65.0	0.0
September	19.0	32.6	25.5	70.0	0.0
October	17.0	30.3	23.1	67.0	0.0
Average	18.4	33.0	25.7	61.2	0.0

### Studied Characteristics

At harvest, plant height (cm) was determined from 10 plants, ear length (cm), weight of kernels/ear (g) number of grains/rows, and number of grains/ears were estimated from 10 ears. 100-kernel weight (g) was determined as an average of three samples, grain yield (t/ha) was estimated from the two middle ridges then converted to tones/ha and nitrogen use efficiency (NUE) was calculated by dividing grain yield by the N rate applied (Moll et al., 1982; Hermanson et al., 2000; Dobermann, 2005).

### Statistical Analysis

The factorial experiment designed in randomized complete block (RCBD) with three replications was used. The first factor was different nitrogen levels, and the second factors was nitrogen application times. The recorded data were analyzed statistically by using statistical software package MSTAT-C (Michigan, 1991). Least Significant Differences (LSD) at 0.05% probability was employed to test the significant differences among means values of each treatments (Torrie, 1996). The two R packages FactoMineR and factoextra were used to generate a principal component analysis (PCA) biplot.

## Results

During the 2019 and 2020 seasons, the results of an analysis of variance (ANOVA) for the two factors, nitrogen rates and application times, as well as their interaction, revealed that nitrogen rates and timing application had a significant at p0.05 and highly significant at p0.01 influence on ear length (cm), plant height (PH), kernels weight/ear (g), 100-kernel weight (g), number of grains/rows, number of grains/ears, grain yield (GY), and nitrogen use efficiency (Tables 3, 4).

**TABLE 3:** ANOVA of the effects of nitrogen rates and times of application and their interaction on plant height, ear length, kernels weight/ear, and 100-kernel weight of maize plants (*Zea mays cv.* SC 10) in both seasons.

Source of variance (SOV)	Degree of freedom (df)	Plant height		Ear length		Kernels weight/ear		100-kernel weight	
		2019	2020	2019	2020	2019	2020	2019	2020
Replicate	2	ns	ns	ns	ns	ns	Ns	ns	ns
A) N-levels	2	**	**	*	*	**	**	**	**
B) N-times	3	**	**	*	*	**	**	**	**
A × B	6	**	**	ns	ns	ns	Ns	ns	ns
Error	18	−	−	−	−	−	−	−	−
CV	−	2.14	2.11	4.7	4.45	5.83	6.01	3.82	3.22
R^2^	−	0.89	0.86	0.86	0.83	0.85	0.84	0.70	0.75
RMSE	−	5.82	5.77	0.80	0.82	1.2	1.9	1.61	1.39

*; **; ns: significant difference at p < 0.05; high significant difference at p < 0.01; not significant difference.

**TABLE 4:** ANOVA of the effects of nitrogen rates and times of application and their interaction on number of grains/rows, number of grains/ears, grain yield (t/ha), and NUE of maize in both seasons.

SOV	df	Number of grains/rows		Number of grains/ears		Grain yield (t/ha)		NUE	
		2019	2020	2019	2020	2019	2020	2019	2020
Replicate	2	Ns	ns	ns	ns	ns	Ns	ns	ns
A) N-levels	2	**	**	**	**	**	**	**	**
B) N-times	3	**	**	*	**	**	**	**	**
A × B	6	**	**	**	**	**	**	**	**
Error	18	−	−	−	−	−	−	−	−
CV	−	3.5	4.4	3.5	3.7	6.4	6.58	8.7	9.28
R^2^	−	0.9	0.8	0.9	0.9	0.8	0.80	0.82	0.81
RMSE	−	1.4	1.8	15.3	16.5	0.3	0.27	1.41	1.47

*; **; ns: significant difference at p < 0.05; high significant difference at p < 0.01; not significant difference.

### Response of Maize Hybrid to Nitrogen Application Levels

The results in Table 5 show that nitrogen fertilizer levels had a significant impact on plant height, ear length, and 100-kernel weight, kernel weight/ear in both seasons (2019 and 2020). In general, the highest values of the characters that contribute to grain yield, such as plant height (286.3 and 285.5 cm), ear length (17.9 and 18.1 cm), kernels weight/ear (100.6 and 103.0 g), and 100-kernel weight (45.8 and 42.2 g), were obtained when nitrogen levels were applied at a rate of 366 kg N/ha. The lowest values were observed at the lowest nitrogen level (192 kg N/ha) of plant height (245.0 and 250.1 cm), ear length (16.7 and 16.9 cm), kernel weight/ear (70.6 and 73.2 g), and 100-kernel weight (39.7 and 39.2 g) in 2019 and 2020 seasons, respectively.

**TABLE 5:** Plant attribute of maize (*Zea mays cv.* SC. 10) as affected by nitrogen levels and time of application in 2019 and 2020 seasons.

Treatments	Plant height (cm)		Ear length (cm)		Kernel weight/ear (g)		100-kernel weight (g)	
	2019	2020	2019	2020	2019	2020	2019	2020
**A-Nitrogen fertilization levels (kg/ha)**								
N_1_	245.0	250.1	16.7	19.6	70.6	73.2	39.7	39.2
N_2_	262.5	265.2	17.0	17.1	85.2	88.5	40.2	39.9
N_3_	276.5	279.1	17.5	17.8	99.1	101.2	44.1	41.0
N4	286.3	285.5	17.9	18.1	100.6	103.3	45.8	42.2
LSD (0.05)	9.5	6.1	0.3	0.2	4.2	4.4	1.4	1.0
**B-Nitrogen fertilization time (split)**								
T_1_	265.8	267.2	16.9	17.0	78.2	84.3	40.1	40.3
T_2_	270.3	275.5	18.4	18.1	85.3	90.5	43.2	43.0
T_3_	275.5	279.3	19.6	19.0	99.1	99.6	44.9	44.1
LSD (0.05)	4.3	3.5	1.1	0.7	1.2	2.5	1.0	1.1

*N1 = nitrogen application at the rate of 192 kg/ha, N2 = 240 kg N/ha, N3 = 288 kg N/ha, and N4 = 366 kg N/ha. T_1_ (50% of N at each of sowing and 1st irrigation), T_2_ (50% of N at each of sowing and 2nd irrigation) and T_3_ (50% of N at each of 1st and 2nd irrigation).*

As shown in Table 6, the highest nitrogen level (366 kg N/ha) produced the highest values of number of grains/row (43.7 and 42.1 grains), number of grains/ear (477.7 and 487.3 grains), and grain yield (4.7 and 4.8 t/ha), followed by 228 kg N/ha, which recorded 4.3 and 4.5 t/ha without any significant differences between these two levels, while the lowest nitrogen level (192 kg N/ha) produced the lowest number of grains/row (38.0 and 39.1 grains), number of grains/ear (417.7 and 425.3 grains), grain yield (3.5 and 3.4 t/ha) during 2019 and 2020 seasons, respectively. Concerning the effect of nitrogen level of NUE, results in Table 6 cleared that NUE significantly affected by changing in applied nitrogen levels in both seasons. It quite clears from these results that NUE was gradually reduced with increasing nitrogen levels where the maximum NUE (22.9 and 19.3) was recorded at 192 kg N/ha, while the lowest NUE (12.6 and 13.1) was obtained at a higher nitrogen level (366 kg N/ha) in 2019 and 2020 seasons, respectively.

**TABLE 6:** Plant attribute of maize (*Zea mays cv.* SC. 10) as affected by nitrogen levels and time of application in 2019 and 2020 seasons.

Treatments	Number of grains/rows		Number of grains/ears		Grain yield (t/ha)		NUE	
	2019	2020	2019	2020	2019	2020	2019	2020
**A-Nitrogen fertilization levels (kg/ha)**								
N_1_	38.0	39.1	417.1	425.3	3.5	3.4	22.9	19.3
N_2_	38.3	39.2	418.6	431.4	3.8	4.1	15.4	17.0
N_3_	40.0	42.1	440.0	458.3	4.3	4.5	13.5	15.6
N4	43.7	44.3	477.7	487.3	4.7	4.8	12.6	13.1
LSD (0.05)	1.4	1.8	15.2	16.3	0.43	0.3	0.8	1.1
**B-Nitrogen fertilization time (split)**								
T_1_	39.0	39.8	428.6	436.1	3.7	3.9	14.0	14.8
T_2_	39.3	40.3	429.1	440.9	4.0	4.2	14.3	16.3
T_3_	42.7	43.6	467.3	474.8	4.3	4.5	16.6	17.5
LSD (0.05)	2.1	1.4	31.5	15.2	0.1	0.3	1.4	0.8

*N1 = nitrogen application at the rate of 192 kg/ha, N2 = 240 kg N/ha, N3 = 288 kg N/ha, and N4 = 366 kg N/ha. T_1_ (50% of N at each of sowing and 1st irrigation), T_2_ (50% of N at each of sowing and 2nd irrigation) and T_3_ (50% of N at each of 1st and 2nd irrigation).*

### Response of Maize Hybrid to Nitrogen Application Time

The results in Table 5 revealed that the timing of nitrogen application had a significant impact on maize grain production and yield contributing characters such as plant height, ear length, and grain weight/ear, 100-kernel. With nitrogen application at T_3_ (50% of N at the first irrigation + 50% of N at the second irrigation), the highest values of plant height (275.5 and 279.3 cm), ear length (19.6 and 19.0 cm), kernel weight/ear (99.1 and 99.6 g) and 100-kernel weight (44.9 and 44.1 g) were attained, followed by T_2_ (50% of N at planting + 50% of N at the second irrigation), which recorded 270.3 and 275.5 cm for plant height, 18.4 and 18.1 cm for ear length, 85.3 and 90.5 g for kernel weight and 43.2 and 43.0 g for weight of 100 kernel, while the lowest values were gotten at T_1_ (50% of N at sowing and 50% of N at first irrigation) in both seasons, respectively.

Results in Table 6, it is clear from these data that nitrogen timing had a significant effect on number of grains/row, number of grains/ear and grain yield/ha, with T_1_ producing the lowest values of number of grains/row (39.0 and 39.8 grains), number of grains/ear (428.6 and 436.1 grains) and grain yield (3.7 and 3.9 t/ha.) and T_3_ producing the highest values of number of grains/row (42.7 and 43.6 grains), number of grains/ear (467.3 and 474.8 grains) and grain yield (4.3 and 4.5 t/ha.), followed by T_2_ with 4.0 and 4.2 t/ha, in the first and second seasons, respectively. Furthermore, the results in Table 6 revealed that T_3_ (50% of N at each of 1st and 2nd irrigation) gave the highest nitrogen utilization efficiency (NUE) values (16.6 and 17.5 percent), whereas T1 (50% of N at each of sowing and 1st irrigation) recorded the lowest (14.0 and 14.8%) in both seasons.

### Response of Maize to the Interaction Between N-Levels and Timing/Splitting

ANOVA for the effect of interaction between nitrogen fertilization rates and the times of application of nitrogen fertilization treatments was shown in Tables 3, 4. The results of ANOVA showed that interaction between nitrogen fertilization levels and the times of application treatments had a highly significant effect on plant height (PH), number of grains/rows, number of grains/ears, grain yield (GY), and nitrogen use efficiency (NUE), while ear length (EL), kernels weight/ear (KW), and 100-kernel weight (g) showed a non-significant result in 2019 and 2020 seasons.

**FIGURE 1:**
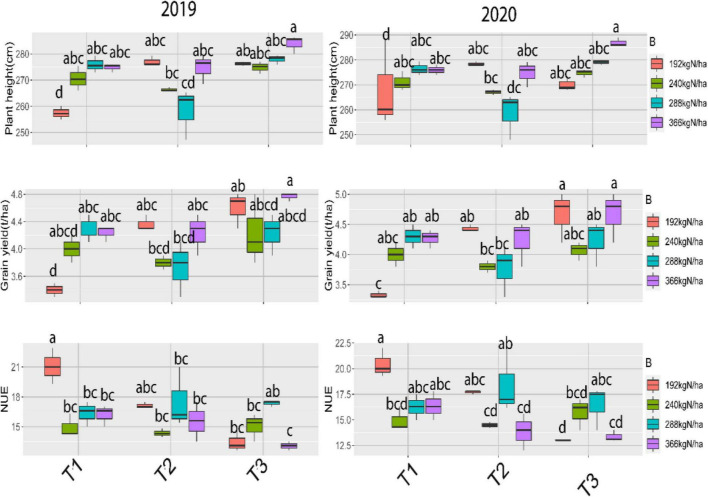
Effects of interaction between nitrogen rates and application time on maize growth parameters across two seasons (2019 and 2020), where T_1_ (50% of N at each of sowing and 1st irrigation), T_2_ (50% of N at each of sowing and 2nd irrigation) and T_3_ (50% of N at each of 1st and 2nd irrigation).

The response of plant height, grain yield, and nitrogen use efficiency (NUE) to the interaction between nitrogen fertilization levels and the time of application (splitting) is shown in the Figure 1. Application of 366 kg N/ha with T3 recorded the highest plants, while the shortest plants of maize were obtained by control treatment in the 2019, and 2020 seasons. Application of 366 kg N/ha with T3 indicated the largest grain yield, also there are no significant differences between 366 and 192 kg N/ha with T3, in contrast, the lowest values of grain yield of maize were recorded by application of 192 kg N/ha with T1 treatment in the first and second seasons, respectively. For NUE, the highest value of NUE was obtained when added 192 kg N/ha along with T1 in both seasons were applied. while T3 with 192 kg N/ha exhibited the lowest NUE in the two seasons.

**FIGURE 2:**
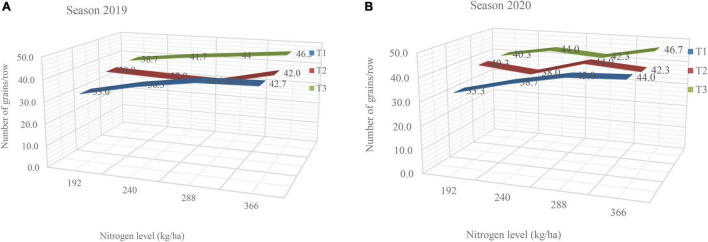
**(A,B)** Interaction effect between N fertilization level and N application time on number of grains/row during the two seasons, where T_1_ (50% of N at each of sowing and 1st irrigation), T_2_ (50% of N at each of sowing and 2nd irrigation) and T_3_ (50% of N at each of 1st and 2nd irrigation).

The interaction effect between nitrogen fertilization levels and the time of application (splitting) on number of grains/row and number of grains/ears is shown in the Figure 2A in the first season and Figure 2B in second season. The results in this figure the maximum mean values of number of grains/row (46.3 and 46.7 grains) were recorded with fertilizing maize plants by the higher rate of the nitrogen with T3 (50% of N at each of 1st and 2nd irrigation) also this rate with T_1_ (50% of N at each of sowing and 1st irrigation) or T_2_ (50% of N at each of sowing and 2nd irrigation) gave the highest numbers in the first and second seasons, respectively.

**FIGURE 3:**
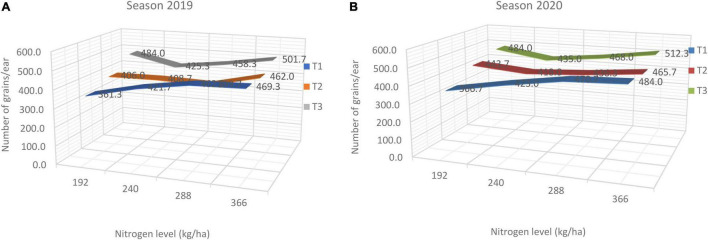
**(A,B)** Interaction effect between N fertilization level and N application time on number of grains/ear in both seasons, where T_1_ (50% of N at each of sowing and 1st irrigation), T_2_ (50% of N at each of sowing and 2nd irrigation) and T_3_ (50% of N at each of 1st and 2nd irrigation).

On the other hand, the interaction effect between nitrogen fertilization levels and the time of application (splitting) on number of grains/ears is shown in the Figures 3A,B in both seasons. The results cleared that applying the higher nitrogen level with the first and second irrigation (T_1_) achieved the highest values of number of kernels/ear (501.7 and 512.3 kernels) in 2019 and 2020, respectively. These results showed that also increasing the rate of N application up to 366 kg/ha with splitting this rate in sowing, or the first or the second irrigation increased number of kernels per row and per ear, in both seasons followed by 288 kg N/ha.

### Principal Component Analysis

**FIGURE 4:**
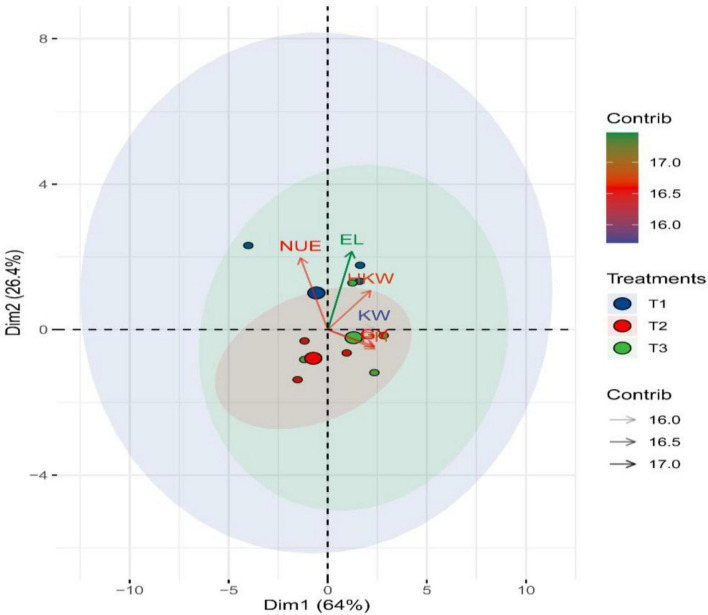
PCA analysis shows the correlations between agronomic traits and NUE traits according to the N fertilization levels and application time over 2 years. Diagrams are defined by the first two axes of the PCA of the different variables (*n* = 3); Axis1 (64% of variance explained) and Axis2 (26.4% of variance explained), where T_1_ (50% of N at each of sowing and 1st irrigation), T_2_ (50% of N at each of sowing and 2nd irrigation) and T_3_ (50% of N at each of 1st and 2nd irrigation).

PCA was performed to study the relationship among the tested traits and treatments (Figure 4). The first two PCs accounted for 90.4% of the variability. PC1 explained 64% of the variation and appeared to be associated with the increase in N level from 192 on the negative side to 360 kg N/ha on the positive side (Figure 4). PC2 accounted for 26.4% of the variation and appeared to be related to the times of application from the top with T3 to the bottom with T1. T3 under 360 kg N/ha was located near grain yield, thus reinforcing the above results.

The angles among trait vectors illustrated the association among the studied traits. Contiguous vectors reflect a strong positive correlation, whereas vectors with large angles (approximately 180°) reveal a negative correlation. A strong positive association was identified between grain yield and all its attributes. The closest components to grain yield were 100-kernel weight and kernel weight/ear, reflecting their importance in indirect selection due to the ease of their measurement. NUE was markedly higher when there was low added 192 N fertilization and were highly correlated with the T1 treatment (Figure 4).

## Discussion

The results revealed that high N application is the most common strategy used by farmers in arid region, to improve productivity. Furthermore, the appropriate timing of N application might maximize maize production, although yield variation is closely related to N rate and external environment. In this line, the highest maize yield was obtained with a N rate of 360 kg/ha in early spring planting and 300 kg/ha in late spring sowing (Coelho et al., 2022). Also, similar results were reported by Yue et al. (2022) they noticed that more than 200 kg N/ha applied one-third at sowing and two-thirds at the six-leaf stage is an appropriate N supply method to improve starch metabolizing enzymes, regulate hormone content, and improve grain-filling rates, thereby increasing maize yield. In the same line, according to Chen et al. (2015), increasing N application (0–240 kg N ha1) clearly boosted yield; however, increasing N application has minimal influence on yield after N application reaches a certain limit. These results are consistent with those obtained by Rong and Xuefeng (2011), Asif et al. (2013), Wasaya et al. (2017) and Hu et al. (2020) who observed that when increasing nitrogen rate was improved the average yield increased in a progressive and positively affected. Additionally, numerous researchers have found that enhancing nitrogen availability has a positive impact on maize productivity (Al-Shebani, 2005; Oraby et al., 2005; Mansour, 2009; Ahmad et al., 2020; Shah et al., 2021b,c).

Grain production improvements as a function of N application rate are explained by increases in the number of kernels/ears, ears/plant, and kernel weight, as well as a decrease in the number of plants lacking ears. The positive response to N fertilizer can be explained by the assumption that nitrogen has the greatest impact on the development of the vegetative parts of the plants, so the beneficial effect of increasing nitrogen supply on yield could be due to better ear growth, more filled kernels per ear, and larger kernels. N increased assimilates supplies for component development and yield set, as evidenced by the significant rise in yield components (Akmal et al., 2010). The influence of nitrogen on the vigor vegetative development and accumulation of photosynthetic assimilates, which create many grains/rows and grains/ear, and meristematic activity of the maize plant, and improving yield qualities such as ultimate grain yield, might explain these results.

In this study, nitrogen fertilization levels and timing increased yield component i.e., number of grain/row, number of grain/ear, grain weight/ear, 100-grain weight, that increased grain yield/ha. N fertilization significantly increased grain yield by enhancing the grain weight, number of ears/ha, and number of grains per ear (Srivastava et al., 2018). The effect of raising the N rate on maize production was stronger in early spring sowing than in late spring sowing, owing to the improved effect of N-fertilizer on kernels per ear (Coelho et al., 2022). The suitable N fertilizer rate and time enhances grain weight as a result of increasing effective grain-filling duration and rate (Abdelsalam et al., 2019; Wei et al., 2019; Hammad et al., 2022; Zhanbota et al., 2022).

Nitrogen fertilizer treatment has a varying maize yield response, and researchers found that applying N with splits resulted in much greater grain production than applying N solely at the base (Abdelsalam et al., 2019; Fabunmi, 2010). These results in this study demonstrated the significance of the development stage at which nitrogen was supplied. This deficit might be caused by the absorption of nitrogen fertilizer ingested by plants destroyed during the thinning process. It is clear from this study that nitrogen application, particularly at T3 and T2, resulted in the highest yield response.

Similarly, Rizwan et al. (2003) reported that applying N in three splits, namely at planting, initial watering, and knee height, resulted in the highest plant height. In comparison to plots where an equal quantity of nitrogen was side dressed in three splits, i.e., at sowing, first irrigation, and knee height, plots where an equal amount of nitrogen was side dressed in three splits yielded the maximum grain yield. These results are consistent with those (Turgut, 2000; Torbert et al., 2001; Nemati and Sharifi, 2012; Tadesse et al., 2013; El-Naggar et al., 2020; Hu et al., 2020) who found that nitrogen application timing/splitting considerably influenced maize grain yield or certain of its components. Grain yield, plant height, ear height, kernel rows per ear, number of kernels/row and per ear, ear length, and thousand grain weight were all significantly affected by split N treatment, according to Adhikari et al. (2016), A rate of 300 kg N/ha generated considerably more grain at 30, 45, 60, and 75 days after planting as compared to control, showing that reducing N losses from the soil and making optimum use of N during crucial growth and development stages of maize would be more cost effective.

In the other study, Wazir and Akmal (2019) observed that three-split N treatment maintained adequate nutrients for maize grain growth, potentially leading to better grain yields. When the N application rate is less than 200 kg N/ha, the results show that N rate, rather than N application time, is the most important factor influencing yield (Li and Li, 2015). Higher N supply, regardless of application timing or plant density, increased grain-fill duration and, more inconsistently, effective grain-filling rate. Kernels accumulated dry matter and N for similar durations (Olmedo Pico and Vyn, 2021). Grain yield is clear by the product of grain number and kernel weight. Although number of kernels is considered the main grain yield determinant because it is more responsive to changes in environmental conditions, grain yield can still be affected by variations in kernel weight (Otegui, 1995; Borrás et al., 2004; Echarte et al., 2006; Kandil et al., 2022).

The overall effectiveness of applied fertilizer is determined by maize absorption of nitrogen from the soil, assimilation, and remobilization into the grain. As a result, uptake and utilization efficiency are two crucial components, and enhancing uptake and utilization efficiency in crops enhances overall NUE (Thomas, 2016; Noor, 2017; Kandil et al., 2020; Congreves et al., 2021). In the present study, applying a higher level of nitrogen (366 kg N/ha) recorded the lowest nitrogen use efficiency (NUE), while the rate of N at (192 kg/ha) recorded the highest NUE. NUE is differed as the extra in harvest grain yield for each increase in applied nitrogen (Good et al., 2004). These results are in harmony with those reported by Zhao et al. (2006), Nemati and Sharifi (2012), and Shemi (2017) who reported that NUE was decreased with increasing nitrogen levels.

Nitrogen-use efficiency (NUE) is a simple estimate of N mass balance. It is defined as the ratio of N eliminated in harvested product divided by the total of N inputs. The effects of other management practices such as N application timing, source and placement are vital for NUE (Power and Schepers, 1989). In our investigation, greater values of all NUE components in lower doses of N resulted from higher use of N absorption in growing grains and reduced N loss to the environment due to synchronization of N delivery timing with crop demands. Several publications suggest that synchronizing N supply with crop demand throughout the crop season is a wonderful way to reduce N loss and increase NUE (Qiu et al., 2015; Srivastava et al., 2018; Aziiba et al., 2019; Gomaa et al., 2021; Chattha et al., 2022). The attained negative relationship between fertilizer rate and NUE (Figure 2) agrees with the results which the rate of applied N is the main factor affecting NUE because N losses increase rapidly when N inputs exceed the crop assimilation capacity (Meisinger and Randall, 1991).

## Conclusion

The following conclusion was formed based on results of this study on the usage of optimal nitrogen rates and timings in future projects, particularly those incorporating modern hybrids. According to the results of the recent field experiments, nitrogen treatments (levels and splits) increase the production and quality of the hybrid maize variety SC 10. All parameters indicated maximum values when nitrogen fertilizer was applied at a rate of 366 kg N/ha, followed by 288 kg N/ha, while the lowest level 192 kg N/ha, followed by 240 kg N/ha, recorded the highest NUE. Combining 366 or 288 kg N/ha with T3 (50 percent N nitrogen fertilizer at first and second irrigation) tends to increase maize hybrid output and yield-related parameters. These findings suggested that Egyptian farmers could improve maize hybrid yield performance by selecting the appropriate nitrogen fertilizer quantity and timing to build a more effective farming cycle with an environmentally friendly or more sustainable system.

## Data Availability Statement

The raw data supporting the conclusions of this article will be made available by the authors, without undue reservation.

## Author Contributions

EG, OE-B, EK, and SL: data curation, investigation, methodology, supervision, and writing – original draft. HA, MS, and NA: software. HA, MS, and JJ: funding acquisition. HA, MS, JJ, OE-B, RG, and NA: writing – review and editing. All authors contributed to the article and approved the submitted version.

## Conflict of Interest

The authors declare that the research was conducted in the absence of any commercial or financial relationships that could be construed as a potential conflict of interest.

## Publisher’s Note

All claims expressed in this article are solely those of the authors and do not necessarily represent those of their affiliated organizations, or those of the publisher, the editors and the reviewers. Any product that may be evaluated in this article, or claim that may be made by its manufacturer, is not guaranteed or endorsed by the publisher.
